# MiRNA-205 modulates cellular invasion and migration via regulating zinc finger E-box binding homeobox 2 expression in esophageal squamous cell carcinoma cells

**DOI:** 10.1186/1479-5876-9-30

**Published:** 2011-03-22

**Authors:** Kayoko Matsushima, Hajime Isomoto, Naoyuki Yamaguchi, Naoki Inoue, Haruhisa Machida, Toshiyuki Nakayama, Tomayoshi Hayashi, Masaki Kunizaki, Shigekazu Hidaka, Takeshi Nagayasu, Masahiro Nakashima, Kenta Ujifuku, Norisato Mitsutake, Akira Ohtsuru, Shunichi Yamashita, Manav Korpal, Yibin Kang, Philip A Gregory, Gregory J Goodall, Shigeru Kohno, Kazuhiko Nakao

**Affiliations:** 1 Department of Gastroenterology and Hepatology, Nagasaki University Hospital, 1-7-1 Sakamoto, Nagasaki 852-8102, Japan; 2 Department of Endoscopy, Nagasaki University Hospital, 1-7-1 Sakamoto, Nagasaki 852-8102, Japan; 3 Department of Pathology, Nagasaki University Hospital, 1-7-1 Sakamoto, Nagasaki 852-8102, Japan; 4 First Department of Surgery, Nagasaki University Hospital, 1-7-1 Sakamoto, Nagasaki 852-8102, Japan; 5 Division of Tumor and Diagnostic Pathology, Atomic Bomb Disease Institute, Nagasaki University Graduate School of Biomedical Sciences, 1-12-4 Sakamoto, Nagasaki 852-8102, Japan; 6 Department of Molecular Medicine, Atomic Bomb Disease Institute, Nagasaki University Graduate School of Biomedical Sciences, 1-12-4 Sakamoto, Nagasaki 852-8102, Japan; 7 Department of Molecular Biology, Princeton University, Washington Road, Princeton, NJ 08544-1014 USA; 8 Hanson Institute and Division of Human Immunology, Institute of Medical and Veterinary Science, Frome Rd, Adelaide, SA 5000, Australia

## Abstract

**Background:**

Esophageal squamous cell carcinoma (ESCC) is often diagnosed at later stages until they are incurable. MicroRNA (miR) is a small, non-coding RNA that negatively regulates gene expression mainly via translational repression. Accumulating evidence indicates that deregulation of miR is associated with human malignancies including ESCC. The aim of this study was to identify miR that could be specifically expressed and exert distinct biological actions in ESCC.

**Methods:**

Total RNA was extracted from ESCC cell lines, OE21 and TE10, and a non-malignant human esophageal squamous cell line, Het-1A, and subjected to microarray analysis. Expression levels of miR that showed significant differences between the 2 ESCC and Het-1A cells based on the comprehensive analysis were analyzed by the quantitative reverse transcriptase (RT)-PCR method. Then, functional analyses, including cellular proliferation, apoptosis and Matrigel invasion and the wound healing assay, for the specific miR were conducted. Using ESCC tumor samples and paired surrounding non-cancerous tissue obtained endoscopically, the association with histopathological differentiation was examined with quantitative RT-PCR.

**Results:**

Based on the miR microarray analysis, there were 14 miRs that showed significant differences (more than 2-fold) in expression between the 2 ESCC cells and non-malignant Het-1A. Among the significantly altered miRs, miR-205 expression levels were exclusively higher in 5 ESCC cell lines examined than any other types of malignant cell lines and Het-1A. Thus, miR-205 could be a specific miR in ESCC. Modulation of miR-205 expression by transfection with its precursor or anti-miR-205 inhibitor did not affect ESCC cell proliferation and apoptosis, but miR-205 was found to be involved in cell invasion and migration. Western blot revealed that knockdown of miR-205 expression in ESCC cells substantially enhanced expression of zinc finger E-box binding homeobox 2, accompanied by reduction of E-cadherin, a regulator of epithelial mesenchymal transition. The miR-205 expression levels were not associated with histological differentiation of human ESCC.

**Conclusions:**

These results imply that miR-205 is an ESCC-specific miR that exerts tumor-suppressive activities with EMT inhibition by targeting ZEB2.

## Background

Esophageal cancer is the eighth most common cancer and the sixth most common cause of cancer deaths worldwide [1]. Although Barrett's adenocarcinoma is the most rapidly increasing cancer in Western countries [2], esophageal squamous cell carcinoma (ESCC) is still dominant in East Asia, including Japan [3]. ESCC is often diagnosed at later stages, so that the prognosis of affected patients is unsatisfactory, despite the development of therapeutic options such as surgery, chemotherapy, and radiotherapy [4]. Consequently, there is a great need for biomarkers to allow a tailored multimodality approach with increased efficacy. To date, nevertheless, efforts to indentify molecular markers in association with the pathogenesis of ESCC have proved to be essentially unsuccessful [5].

MicroRNAs (miRs) are small, non-coding RNAs that negatively regulate gene expression via translational repression or messenger RNA degradation. More than 700 miRs have been identified and registered in humans, with each individual miR predicted to target multiple genes based on the seed sequence matches in their 3'-untranslated regions (UTRs) [6]. MiRs are involved in biological and pathologic processes, including cell differentiation, proliferation, apoptosis, and metabolism [7], and they are emerging as highly tissue-specific biomarkers with potential clinical applicability for defining cancer type and origin [8,9]. Accumulating evidence indicates that deregulation of miRs is associated with human malignancies and suggests a causal role of miRs in tumor initiation and progression, since they can function as oncogenes or tumor suppressors [10]. In fact, previous studies showed distinct differences in miR expression patterns between squamous cell carcinoma and adenocarcinoma in esophageal and other cancers [3,11,12]. Kimura *et al. *reported that miR-205 showed highest expression in both benign and malignant squamous epithelia including ESCC, although it was less expressed in cell lines and tissues other than squamous epithelia. On the other hand, miR-21, which is an oncogenic miRNA in various malignancies, was also up-regulated in ESCC compared to paired normal squamous epithelia [13]. However, there has been little information on the functional roles of miRs specific for ESCC [14].

Epithelial to mesenchymal transition (EMT) describes the molecular reprogramming and phenotypic changes involved in the conversion of polarized immotile epithelial cells to motile mesenchymal cells [15]. EMT occurs during fundamental biological and disease processes including development and cancer [16]. EMT in cancer leads to the loss of cell-cell adhesion and cell polarity as well as altered cell-extracellular matrix interactions, resulting in invasion and metastasis [16]. E-cadherin is a central component of the adherens junction complex responsible for calcium dependent cell-cell adhesion and maintenance of cytoskeletal organization [15,16]. Loss of E-cadherin expression can be a common marker of EMT and has been identified as a causal factor in cancer progression [15,16]. Transcriptional repression of the E-cadherin gene is emerging as an important mechanism through which E-cadherin is downregulated during tumor progression and such factors as snail, slug/snail2, zinc finger E-box binding homeobox (ZEB) 1 and ZEB2 have been shown to directly bind to the E-cadherin promoter and repress its transcription [15]. Several recent studies have identified miR-200 family as key regulators of EMT and enforcers of the epithelial phenotype [17,18]. In fact, the miR-200 family participates in a signaling network with the E-cadherin transcriptional repressors ZEB1 and ZEB2. Using microRNA target prediction algorithms, ZEBs were predicted to contain multiple sites for miR-200 family and in reporter assays their 3'UTR was functionally responsive to the manipulation [15-17]. In addition, two miR-205 binding sites were indentified in ZEB2 [15,17], suggesting EMT could be also regulated by miR-205. The present study was designed to identify miRs that could be specifically expressed and exert distinct biological actions in ESCC cells.

## Methods

### Cell lines and cultures

I) Five cell lines of human ESCC cells (OE21, TE5, TE8, TE10, and TE11), a non-malignant human esophageal squamous cell line immortalized by SV40 infection, Het-1A, 2 human Barrett's adenocarcinoma cell lines (Bic-1 and Seg-1), 3 human gastric adenocarcinoma cell lines (AGS, AZ521 and KATOIII), 2 colorectal adenocarcinoma cell lines (Caco-2 and DLD1), a human cervix epithelioid carcinoma cell line (HeLa), a human lung adenocarcinoma cell line (A549), and human hematological malignant cell lines (acute promyelotic leukemia, HL60; human T cell lymphoblast-like cell line, Jurkat; and histiocytic lymphoma, U937) were cultured. The AZ521, KATOIII, DLD-1, HeLa, A549, HL60, and U937 cells were purchased from the Japanese Collection of Research Bioresources Foundation (Sennan, Japan). The OE21, Het-1A, AGS, and Caco-2 cells were obtained from the American Type Culture Collection (Manassas, VA). The TE5, TE8, TE10, and TE11cells were purchased from Riken Bioresource Center Cell Bank (Tsukuba, Japan). Bic-1 and Seg-1were kindly provided by Dr. D.G. Beer (Department of Surgery, Section of General Thoracic Surgery, Michigan Medical School, Ann Arbor, MI). The OE21, TE5, TE8, TE10, TE11, Het-1A, U937, HL-60, DLD-1, Jurkat, and KATOIII cells were grown in RPMI 1640 medium, while the HeLa, A549, and Caco-2 cells were maintained in Dulbcco's modified Eagle medium. Both media were supplemented with 10% fetal bovine serum, 1% penicillin/streptomycin, and 1% glutamine, and all cell lines were cultured in a humidified incubator with 5% CO_2 _at 37°C.

### Patients and Clinical samples

ESCC patients who underwent esophagoscopy between June 2007 and December 2010 were recruited. After obtaining informed consent, 3 biopsy samples each were taken from the ESCC tumor and the matched normal-appearing surrounding esophageal mucosa under endoscopic observation. Two of these samples were placed immediately into 1 mL of RNAlater (Applied Biosystems, Foster City, CA) for RNA isolation later. The other specimen was fixed in 10% formalin and embedded in paraffin for histopathology. The paraffin-embedded biopsy specimens were cut into 5-μm-thick sections and stained with hematoxylin and eosin, and the three pathologists (T.N., M.N., and T. H.) classified the ESCC differentiation.

### RNA extraction

Total RNA including miR from the tissue samples and cultured cells was extracted using a commercial kit (mirVana RNA™ Isolation kit, Applied Biosystems) according to the supplier's instructions. Quality of total RNA was determined on a Bioanalyzer (Bioanalyzer RNA Nano kit, Agilent, Santa Clara, CA), and the RNA was quantified using a Nanodrop-1000 spectrophotometer (Nanodrop Technologies, Wilmington, DE). Extracted RNA samples were stored at -80°C until used.

### MiR array hybridization and analysis

To find specific miR(s) for ESCC cells, total RNA was extracted from OE21 and TE10 cells, representative well and moderately differentiated human ESCC cell lines, respectively, and the non-malignant human esophageal squamous cell line, Het-1A. The isolated RNA samples were subjected to comprehensive analysis of miRNA expression patterns with the microarray-based technology, an Agilent Human miRNA array chip version 1 (Agilent), containing 15,000 probes corresponding to 470 unique human miRs and 64 human viral miRs cataloged in the Sanger database version 9.1. One hundred ng of each total RNA aliquot were treated with calf intestine phosphatase (GE Healthcare, Chalfont St Giles, UK), denatured using DMSO (Sigma, St Louis, MO), and directly labeled with Cy3 using T4 RNA ligase (GE Healthcare). Labeled samples were hybridized to the miR array 8 × 15 k (G4470A) platforms in SureHyb chambers (Agilent), washed with the buffer supplied (Agilent), according to the manufacturer's instructions, and scanned using an Agilent Scanner (G2505B). Data were extracted using Feature Extraction Software 9.3 and GeneSpring software (Agilent). To identify miRs that were differentially expressed between the ESCC cell lines and Het1A cells, supervised analysis was performed using significance analysis of microarrays (SAM, Stanford University, Stanford, CA). The differences in miR expressions were considered significant if the fold change of expression values was >2.0 and the p value was < 0.05 using the *t*-test.

### Quantitative reverse transcription-polymerase chain reaction (RT-PCR) analysis for miRs

Expression levels of miRs that showed significant differences based on the microarray results were analyzed by quantitative RT-PCR using various human malignant cell lines including ESCC and non-malignant Het-1A. cDNA was prepared from total RNA using a TaqMan MicroRNA Reverse Transcription Kit (Applied Biosystems). Predesigned TaqMan MicroRNA Assays including the primer set and TaqMan probe were purchased from Applied Biosystems. The reverse transcription reactions were performed in aliquots containing 50 ng total RNA,1.5 μl 1 × RT Primer, 1 μl 10 × RT Buffer, 0.15 μl 100 mM dNTP,1 μl reverse transcriptase, and nuclease-free water added up to 15 μl at 16°C for 30 min, followed by 42°C for 30 min and 85°C for 5 min. All PCR reactions were performed in 20-μl aliquots containing 1.33 μl miR RT products with 18.67 μl PCR master mixture (10 μl 2 × Universal PCR master mix, 1 μl each primer, 1 μl Taqman Probe, and 6.67 μl nuclease-free water), and run in triplicate on the 7500 Real-Time PCR system (Applied Biosystems). Thermal cycling was initiated with a first denaturation step at 95°C for 10 min, followed by 40 cycles of 95°C for 15 s and 60°C for 1 min. The cycle passing threshold (Ct) was recorded for each candidate miR, and a small RNA, U6B, was used as the endogenous control for data normalization. Relative expression was calculated using the formula 2^-^^DCt ^= 2^-^^(Ct, U6B ^^- ^^Ct,Specific) ^as described in the ABI PRISM 7700 SDS relative quantification of gene expression protocol by PE Applied Biosystems. Similarly, total RNAs extracted from the neoplastic and non-neoplastic samples (esophagoscopic biopsies) were subjected to real-time quantitative RT-PCR for quantitation of miR-205 expression levels.

### Northern blot analysis

Ten micrograms of total RNA were separated on 15% denaturing polyacrylamide gel and electrotransferred onto Nylon Membrane Positively Charged (Roche Diagnostics, Basel, Switzerland). Oligonucleotides complementary to mature miR-205 were labeled with digoxigenin by terminal transferase-mediated 3' end-labeling and used as probes. The sequence of oligonucleotides was 5'-cagactccggtggaaatgaagga-3'. The membrane was then hybridized with hybridization mixture (0.25 M Na_2_HPO_4 _[pH 7.2], 1 mM ethylenediamine tetraacetic acid (EDTA), 1% bovine serum albumin, 7% sodium dodecyl sulfate (SDS), 15% formamide, and the labeled probe) overnight at 43°C. After hybridization, the membrane was washed with wash mixture (20 mM Na_2_HPO_4 _[pH 7.2], 1 mM EDTA, 1% SDS) followed by the washing buffer (0.1 M maleic acid, 0.15 M NaCl, 0.3% Tween-20). After blocking with 1% Blocking Reagent (Roche Diagnostics), the hybridized membrane was incubated with alkaline phosphatase-conjugated anti-digoxigenin antibody (Roche Diagnostics). The membrane was then washed with the washing buffer. After equilibration with the detection buffer (0.1 M Tris-HCl [pH 9.5], 0.1 M NaCl), the membrane was incubated with the chemiluminescent substrate CDP Star (Roche Diagnostics). Detection was performed using a LAS3000 imaging system (Fujifilm, Tokyo, Japan).

### Western blot

Cultured cells were directly lysed for 30 minutes on ice with lysis buffer [50 mmol/L Tris-HCl (pH 7.4), 1% Nonidet P-40, 0.25% sodium deoxycholate, 150 mmol/L NaCl, 1 mmol/L EDTA, 1 mmol/L PMSF, 1 μg/mL aprotinin, 1 μg/mL leupeptin, 1 μg/mL pepstatin, 1 mmol/L Na_3_VO_4_, and 1 mmol/L NaF]. After centrifugation at 13,000 g for 15 minutes, protein concentrations were measured using Bradford's reagent (Bio-Rad laboratories, Hercules, CA), and protein was denatured by boiling for 10 minutes. Protein (25 μg) was loaded onto sodium dodecyl sulfate-polyacrylamide gels for electrophoresis and then transferred onto nitrocellulose membranes. After blocking with 5% milk in TBST (137 mmol/L NaCl, 25 mmol/L Tris, and 1 mmol/L disodium ethylenediaminotetraacetate containing 0.1% Tween-20), the membranes were incubated with mouse monoclonal anti-E-cadherin (1:1000, BD Biosciences, Franklin Lakes, NJ) and anti-N-cadherin (1:1000, BD Biosciences), and rabbit anti- ZEB1 (1:200, Santa Cruz Biotechnology, Santa Cruz, CA), anti-ZEB2 (1:200, Santa Cruz Biotechnology), anti-phospho (Ser473)-Akt (1:500, Cell Signaling Technology, Tokyo, Japan) and anti-β-actin (1: 1000, Santa Cruz Biotechnology) at 4°C overnight. After washing with TBST 3 times (10 minutes each), the membranes were incubated with their corresponding horseradish peroxidase (HRP)-conjugated secondary antibodies at room temperature for 1 hour. After washing with TBST 3 times (10 minutes each), bound antibodies were visualized using enhanced chemiluminescent substrates (Amersham, Arlington Heights, IL).

### MiR-205 precursor and anti-miR-205 inhibitor transfection

The OE21 cells were seeded (8 × 10^5 ^cells in 4 ml of RPMI1640 per dish) in 60-mm culture dishes and grown overnight. Transfection of miR-205 precursor, anti-miR-205 inhibitor, or each negative control (all purchased from Applied Biosystems) at indicated concentrations was introduced into the cell using 20 μl siPort NeoFX Transfection Agent (Applied Biosystems) in 500 μl Opti-MEM (GIBCO™, Invitrogen, Carlsbad, CA) according to the manufacturer's recommendations. The negative controls were scrambled oligonucleotides that were validated not to produce identifiable effects on known miR function (http://www.ambion.com/jp/catalog/ProdGrp.html?fkProdGrp=344, http://www.ambion.com/catalog/CatNum.php?17100). We confirmed successful transfections using real-time RT-PCR for miR-205.

### Cell proliferation assay

Cellular proliferation was assessed by the 3-(4,5-dimethylthiazol-2-yl)-5-(3-carboxymethoxyphenyl)-2-(4-sulfophenyl)-2H-tetrazolium (MTS) assay (Promega, Madison, WI). OE21 cells were plated at a density of 3 × 10^3 ^cells/well on 96-well plates and grown overnight. For each well, anti-miR-205 inhibitor molecule, miR-205 precursor, or each scrambled negative control was introduced into each well at a concentration of 50 nM. Twenty-four hours later, the assay was initiated by adding 20 μL of MTS solution reagent to 100 μL of culture medium for each well. After incubation for 3 hours at 37°C, the plates were read in a microplate autoreader (Molecular Devices, Sunnyvale, CA) at wavelength of 490 nm. The results were expressed as the mean optical density for selected paradigms performed in duplicate.

### Quantitation of apoptosis

OE21 cells were plated in 12-well plates at a density of 1 × 10^5^cells per well and incubated overnight. Then, 50 nM anti-miR-205 inhibitor, miR-205 precursor, or each scrambled negative control was transfected. Twenty-four hours later, apoptosis was quantitated by assessing the characteristic nuclear changes of apoptosis (i.e., chromatin condensation and nuclear fragmentation) using fluorescence microscopy (Eclipse TE200; Nikon Instruments, Melville, NY) after DAPI (4',6'-diamidino-2-phenylindole dihydrochloride, Roche Diagnostics) staining at a concentration of 10 μg/mL for 15 minutes, as previously described [19].

### Transwell invasion assay

OE21 cells were seeded at a density of 2.0 × 10^6^/well on 60-mm Petri dishes, and 24 hours later, the cells were transfected with either 50 nM anti-miR-205 inhibitor or scrambled negative control. After 24 hours, the transfected cells were harvested by trypsinization, and washed twice in PBS, and 2.5 × 10^4 ^cells were transferred to the upper chamber, a BioCoat™ Matrigel™ Invasion Chamber (BD Biosciences) with inserts containing an 8-μm-pore-sized membrane with a thin layer of Matrigel in the 24-well Transwell plate filled with 500 μL serum-free RPMI1640 medium. In the lower chamber, 750 μL of the 10% FBS-containing medium were added. After incubation for 24 hours, the invaded cells were counted under microscopic observation using a Diff-Quick staining kit (Sysmex, Kobe, Japan ).

### Wound healing assay

OE21cells were transfected with either 50 nM anti-miR-205 inhibitor or scrambled negative control. When cell confluence reached about 80% at 48-hours post transfection, wounds were created in confluent cells using a 200-μl pipette tip. The cells were then rinsed with medium to remove any free-floating cells and debris. Medium was then added, and culture plates were incubated at 37°C. Wound healing was observed at different time points within the scrape line, and representative scrape lines were photographed. Duplicate wells for each condition were examined, and each experiment was repeated three times.

### ZEB1 and ZEB2 3'-UTR luciferase reporter assays

The 3'-UTRs for both ZEB1 and ZEB2 were PCR-amplified from genomic DNA as described previously [18]. The Amplified 3'-UTRs were cloned downstream of the firefly luciferase coding region in the pMIR-REPORT™ (Applied Biosystems). OE21 cells were seeded in 24-well plates 24 hours prior to transfection. The following day, 200 ng of reporter plasmid along with 200 ng of control Renilla-luciferase plasmid were co-transfected using FuGENE^® ^(Roche Diagnostics). Cells were collected 24 hours after transfection and assayed for luciferase activity using the Glomax 96 luminometer (Promega). To assess the effect of miR-205 on reporter activity, either 50 nM of miR-205 precursor (Applied Biosystems) or the negative control was co-transfected.

### Statistical analysis

The differences between groups were analyzed using the unpaired, one-tailed, Student's *t-*test. Data were expressed as means ± standard error. Differences were considered statistically significant at *p *< 0.05. All examinations were conducted according to Good Clinical Practice and the Declaration of Helsinki, and they were approved by the Nagasaki University ethics committees.

## Results

### miR-205 is specifically upregulated in ESCC cells

**Figure 1:**
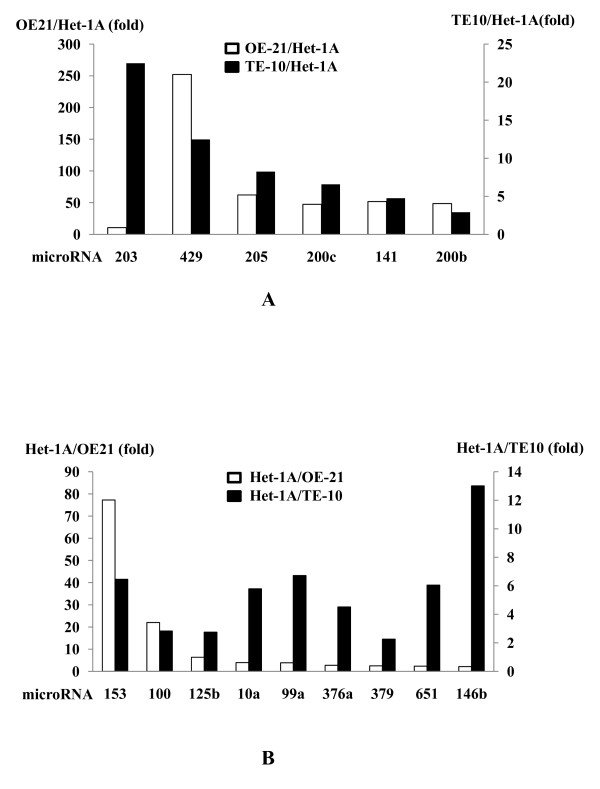
**The comparison of miRNA profile in ESCC cell lines (OE21 or TE10) and non-ESCC cell line (Het1A)**. MicroRNA (miR) microarray showed that miR-203, -429, -205, -200c, and -141 were significantly (more than 2-fold) overexpressed in the 2 esophageal squamous cell carcinoma (ESCC) cell lines, OE21 (while bars) and TE10 (black bars), compared to the non-malignant esophageal squamous cell line, Het1A cells (A). On the other hand, miR-153, -100, -125b, -10a, -99a, -376a, -379, -651, and -146b were significantly reduced in expression in both ESCC cell lines compared to Het-1A cells (B).

**Figure 2:**
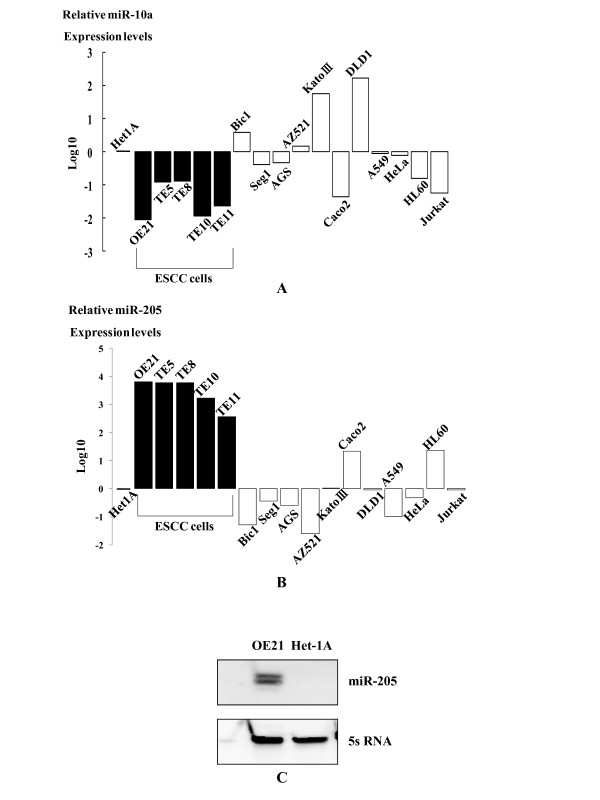
**MiRNA-10a and miR-205 expression levels in various malignant cell types**. Quantitative reverse transcriptase (RT)-PCR revealed that the miR-10a expression levels were decreased in ESCC cell lines (OE21, TE5, TE8, TE10, and TE11) compared to Het1A cells but the other cell lines (Caco2 and Jurkat) had more decreased expression (A). On the other hand, the miR-205 expression levels were exclusively increased in each ESCC cell line compared to those in any other malignant cell types examined and Het-1A cells (B). Northern blot analysis showed the intense miR-205 expression in OE21 cells despite its nominal expression in Het-1A cells (C).

Based on the miR microarray analysis, miR-203, -429, -205, -200c, and -141 were significantly (more than 2-fold) overexpressed in both ESCC cell lines compared to non-malignant Het-1A cells (Figure 1A). On the other hand, miR-153, -100, -125b, -10a, -99a, -376a, -379, -651, and -146b were significantly lower in expression in the two ESCC cell lines than in Het-1A cells (Figure 1B). Thus, real-time RT-PCR was used to quantify expression levels of miRs that showed significant alterations on the microarray analysis. Among the significantly altered miRs, only the miR-205 and -10a expression levels were substantially increased and decreased, respectively, in all ESCC cell lines (OE21, TE5, TE8, TE10, and TE11) compared to Het-1A cells on quantitative RT-PCR (Figure 2A, 2B). Indeed, the miR-10a expression levels were decreased in ESCC cell lines (OE21, TE5, TE8, TE10, and TE11) compared to Het-1A cells but the other cell lines (Caco-2 and Jurkat) had more decreased expression (Figure 2A). On the other hand, the miR-205 expression levels are exclusively increased in each ESCC cell line compared to those in any other malignant cell types examined and Het-1A cells (Figure 2B). Northern blot analysis shows the intense miR-205 expression in OE21 cells despite its nominal expression in Het-1A cells (Figure 2C). These results indicate that overexpression of miR-205 could be specific to ESCC cells, and hence, we sought to determine the functional roles of miR-205 in ESCC.

### miR-205 is not involved in cellular proliferation or apoptosis of ESCC

**Figure 3:**
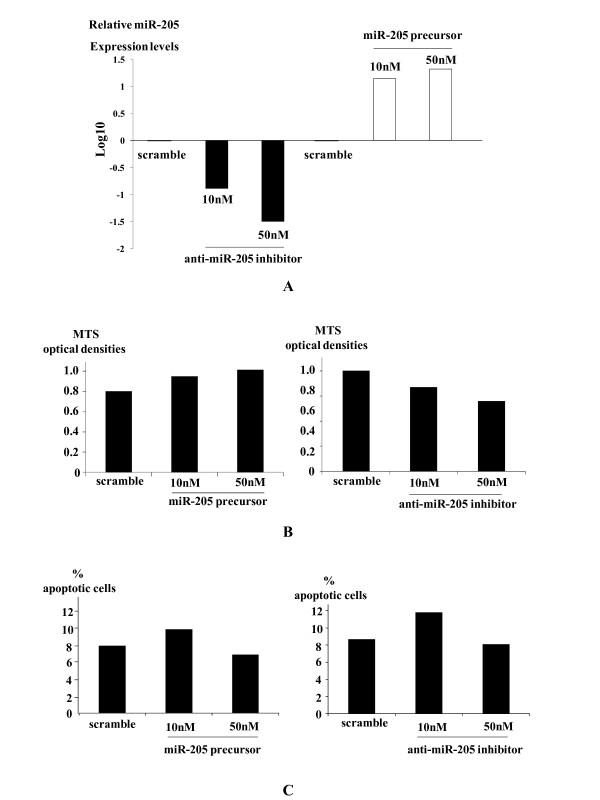
**MiR-205 is not involved in cellular proliferation or apoptosis of ESCC**. Transfection of miR-205 precursor or anti-miR-205 inhibitor with sufficient concentrations substantially increased or decreased miR-205 expression levels in OE21 cells, respectively, assessed by quantitative RT-PCR (A). There were no significant differences between OE21 cells transfected with miR-205 precursor and anti-miR-205 inhibitor at the indicated concentrations in the optical densities of 3-(4,5-dimethylthiazol-2-yl)-5-(3-carboxymethoxyphenyl)-2-(4-sulfophenyl)-2H-tetrazolium (MTS) assays (B). There were no significant differences in the percentages of apoptotic cells with morphological characteristics between the OE-21 cells transfected with miR-205 precursor and anti-miR-205 inhibitor at the indicated concentrations (C).

Transfection of miR-205 precursor or anti-miR-205 inhibitor with sufficient concentrations to increase or decrease miR-205 expression levels, respectively (Figure 3A), had no significant impact on the optical densities of MTS assays (Figure 3B). Again, there were no significant differences in the percentages of apoptotic cells between the OE21 cells transfected with 50 nM miR-205 precursor and anti-miR-205 inhibitor (Figure 3C).

### miR-205 modulates cellular invasion and migration of ESCC

**Figure 4:**
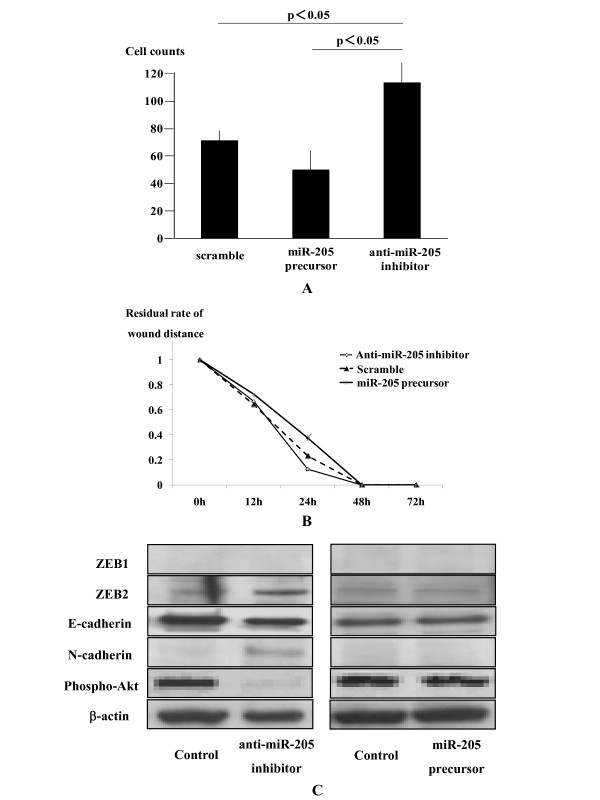
**MiR-205 reduces epithelial-mesenchymal transition(EMT) through regulating zinc finger E-box binding homeobox2 (ZEB2) expression**. Knockdown of miR-205 by transfection with 50 nM anti-miR-205 inhibitor significantly increased the invaded cell numbers on the Matrigel invasion assay as described in Materials and Methods, while overexpression of miR-205 by 50 nM miR-205 precursor transfection significantly inhibited the transmembrane ability compared to control scramble oligonucleotides (A). Transfection with 50 nM miR-205 precursor significantly inhibited the distance of OE21 cell migration, while transfection with 50 nM anti-miR-205 inhibitor tended to promote *in vitro *wound healing as described in Materials and Methods, though it is not significant (B). Western blot showed that knockdown of miR-205 by 50 nM anti-miR-205 inhibitor transfection leaded to enhanced expression of zinc finger E-box binding homeobox (ZEB) 2 but not ZEB1 in OE21 cells (C). The downregulation of miR-205 decreased cellular E-cadherin expression, and instead, N-cadherin appeared in the OE21 cells transfected with anti-miR-205 inhibitor. Overexpression of miR-205 by its precursor (50 nM) did not affect the expression levels of ZEBs and E- and N-cadherin. Transfection of anti-miR-205 inhibitor but not miR-205 precursor reduced cellular expression of phospho-Akt (C).

Knockdown of miR-205 by transfection with anti-miR-205 inhibitor significantly increased the invaded cell numbers on the Matrigel invasion assay, while overexpression of miR-205 by miR-205 precursor transfection significantly inhibited the transmembrane ability (Figure 4A). Consistent with the results of the *in vitro *Matrigel invasion assay, transfection with miR-205 precursor significantly inhibited the distance of OE21 cell migration, while transfection with anti-miR-205 inhibitor tended to promote *in vitro *wound healing, though it was not significant (Figure 4B).

### miR-205 induces an epithelial-mesenchymal transition (EMT)-like phenotype through regulating zinc finger E-box binding homeobox 2 (ZEB2) expression

Consistent with this, knockdown of miR-205 by anti-miR-205 inhibitor transfection enhanced cellular expression of ZEB2 but not ZEB1 in OE21 cells (Figure 4C). On the other hand, overexpression of miR-205 by its precursor did not have impact on the expression of ZEBs. Downregulation of miR-205 decreased cellular E-cadherin expression, and instead, N-cadherin appeared in the OE21 cells transfected with anti-miR-205 inhibitor (Figure 4C), indicating acquisition of the EMT-like phenotype [16]. Overexpression of miR-205 by its precursor did not affect the expression levels of E- and N-cadherin. Again, transfection of anti-miR-205 inhibitor but not miR-205 precursor reduced cellular expression of phospho-Akt, consistent with recent studies [20,21].

### miR-205 directly targets ZEB2

**Figure 5:**
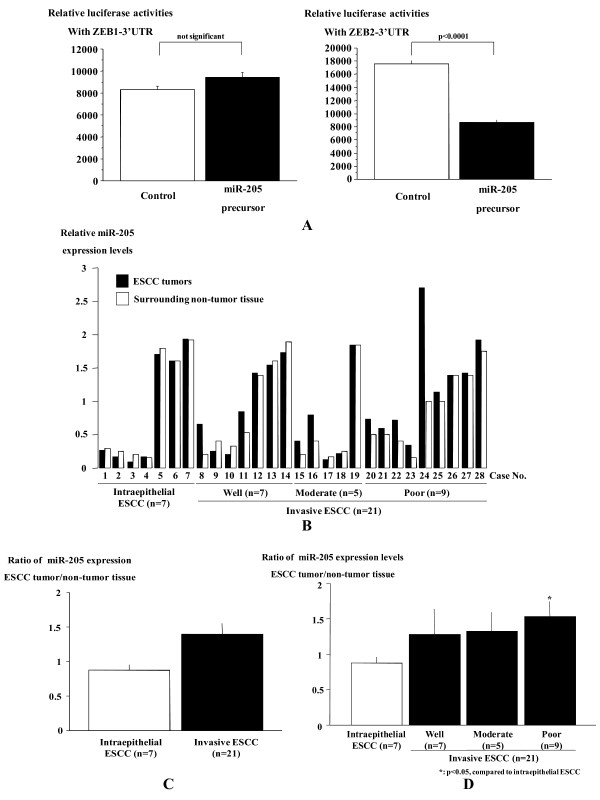
**MiR-205 directly targets ZEB2 and miR-205 expression level in invasive ESCC tumors with poor differentiation is higher than in intraepithelial ESCC tumors**. Activities of the firefly luciferase with the ZEB1 or ZEB2 3'-untranslated region (UTR) in the presence of co-transfected negative control (white bar) or miR-205 precursor (black bar). The luciferase activities were shown as the ratio of firefly to Renilla luciferase activity and measured after 24 h in triplicates (A). The miR-205 expression levels in ESCC tumor samples and matched non-cancerous surrounding mucosa of the esophagus were measured using quantitative RT-PCR. There are no significant difference in the miR-205 expression levels between the ESCC tumors (white bars) and their paired surrounding non-tumor tissues (black bars), though miR-205 is highly expressed in the tumors of 16 of 28 cases examined (B). No significant difference was observed between intraepithelial and invasive ESCC samples (C). The miR-205 expression levels did not differ among the histological subclasses of ESCC differentiation, but invasive ESCC with poor differentiation showed more significantly increased expression of miR-205 than intraepithelial ESCC (D).

Co-transfection of the reporter plasmid along with miR-205 precursor resulted in a significantly reduced ZEB2-3'-UTR-luciferase expression, suggesting that miR-205 is likely to target ZEB2 directly (Figure 5A). In reporter assay using the ZEB1 3'-UTR, however, miR-205 precursor was unable to reduce the luciferase reporter expression (Figure 5A).

### miR-205 is not involved in cellular differentiation of ESCC tumors

There were 7 intraepithelial and 21 invasive ESCC patients. The invasive ESCCs were composed of each 7 well, 5 moderate and 9 poor differentiation, respectively. The miR-205 expression in ESCC tumor samples was assessed using real-time RT-PCR. There were no significant differences in the relative miR-205 expression levels between the ESCC tumors and their paired surrounding non-tumor tissues, though miR-205 was highly expressed in the tumors of 16 of 28 cases examined (Figure 5B). The miR-205 expression did not differ significantly between intraepithelial and invasive ESCC samples. The miR-205 expression levels did not differ among the histological subclasses of ESCC differentiation (Figure 5C), albeit those in invasive ESCC with poor differentiation were significantly lower than in intraepithelial ESCC (Figure 5D).

## Discussion

Several studies showed the differentially expressed miRs in human ESCC tissues [3,11,13,14,22-28] (Table 1). In the present study, miR-205 was exclusively overexpressed in ESCC. The miR-205 expression levels were higher in ESCC cells than in any of the other cell lines derived from different malignancies. In most clinical cases of ESCC, miR-205 expression was more enhanced in ESCC tumors than in the paired non-cancerous esophageal mucosa. It has been reported that miR-205 could be a discriminator between esophageal squamous and metaplastic epithelium (Barrett's esophagus) [11]. Tran *et al *conducted profiling of miR expression in human head and neck squamous cancer cell lines, and they detected 33 highly and 22 lowly expressed miRs. Among them, miR-205 and -212 were listed among the highest miRs in expression [29]. Another study identified miR-205 as one of a set of 6 miRs that were differentially expressed in pulmonary squamous cell lung carcinoma compared to adenocarcinoma [30]. These data are in agreement with previous reports that miR-205 was abundant in squamous cells in humans [30,31]. MiR-205 is a highly conserved miR with homologs in diverse species [30,32,33]. In zebra fish, miR-205 is predominantly expressed in the epidermis, while in mice, it was detected in the footpad, tongue, epidermis, and corneal epithelium, but not in the small intestine, brain, heart, liver, kidney, and spleen [5,32,33]. These observations suggest that miR-205 might represent a stratified squamous epithelium miR.

**Table 1:** A list of the differentially expressed microRNA (miR)s in human esophageal squamous cell carcinoma tissues in the literatures and in our study

Overexpression	Down-regulation
miR-21	miR-30a-3p
miR-92a	miR-133a
miR-93	miR-133b
miR-129	miR-145
miR-205	miR-203
miR-296	miR-210
miR-373	miR-375

On the other hand, miR profiling revealed that miR-205 expression was downregulated in some other type of malignancies, such as breast and prostate cancer [34-36]. Iorio et al reported that miR-205 was significantly underexpressed in breast tumors compared with matched normal mammary tissue. Furthermore, breast cancer cell lines expressed lower levels of miR-205 than the non-malignant mammary cells examined in their study [34]. Of note, ectopic expression of miR-205 significantly inhibited cell proliferation and anchorage-independent growth in breast cancer cells, possibly via targeting HER (human epidermal growth factor receptor) [34]. In this context, miR-205 could interfere with the phosphatidylinositol-3 kinase/Akt survival pathway mediated by HER [34]. Although miR-205 did not affect cellular proliferation, apoptosis, and differentiation of ESCC in the present study, knockdown of miR-205 significantly promoted the locomotion and invasion of ESCC cells. This is the first study that involved functional analyses of a specific miR for ESCC.

Similar to our observations, miR-205 was found to function as a tumor suppressor in diverse cell types [34-37]. Enforced expression of miR-205 was shown to inhibit cell invasion and suppress lung metastasis of breast cancer cells in nude mice, possibly through targeting ErbB3 [35]. MiR-205 also exerts inhibitory effects on cellular invasiveness and migration in prostate cancer and glioblastoma cells, through down-regulation of the protein kinase Cε and low-density lipoprotein receptor-related protein 1, respectively [36,37]. Using miR target prediction algorithms, ErB3, E2F1, E2F5, ZEB1, ZEB2, and protein kinase Cε have been indentified as putative miR-205 targets [36]. In the present study, knockdown of miR-205 expression substantially enhanced cellular expression of ZEB2 in ESCC cells. In fact, previous and present studies employing a reporter gene assay confirmed miR-205 binding to the ZEB2 3'-UTR [15,17]. Although the ESCC cells examined in this study did not express ZEB1 sufficiently, direct interaction of miR-205 with ZEB1 3'-UTR was shown in other cell types but not in ESCC cells examined in this study [15,17]. ZEB1 and ZEB2 are related homeodomain-containing transcriptional factors that repress E-cadherin transcription [17,38,39]. E-cadherin is a central component of the adherens junction complex responsible for cell-cell adhesion and maintenance of cytoskeleton organization [15]. It is known that loss of E-cadherin expression is a key event in the EMT, which can be recapitulated during tumor progression, constituting an early step in tumor metastasis including ESCC [15,40-43]. In line with this, cellular E-cadherin expression was substantially reduced, whereas N-cadherin expression emerged in ESCC cells transfected with anti-miR-205 inhibitor to suppress ZEB2, and they were endowed with properties allowing augmented invasion through EMT. Gregory *et al *described that the miRNA-200 family (miR-200a, miR-200b, miR-200c, miR-141, and miR-429), as well as miR-205, was markedly downregulated in breast and colon cancer cells that had undergone EMT [15]. Collectively, miR-205, along with members of the miR-200 family, can be a key regulator of EMT to widely enforce the indolent epithelial-like phenotype, not limited to ESCC. In clinical settings, lower levels of miR-205 were significantly associated with loco-regional recurrence and poor survival of patients with head and neck squamous cell carcinoma [41]. Further studies are warranted to assess whether miR-205 expression levels could be a predictive biomarker for clinical outcomes in ESCC.

## Conclusions

MiR-205 expression was specifically increased in ESCC cells. MiR-205 is likely to control cell invasion and migration in ESCC cells through its repression of ZEB2, a repressor of E-cadherin. These findings establish the tumor-suppressive role of miR-205, which may serve as a unique therapeutic target for ESCC.

## Competing interests

The authors declare that they have no competing interests.

## Authors' contributions

KM and HI participated in the design of the study, worked up the ESCC cases; supported data analysis and drafted the manuscript. NK was involved in study design and drafted the manuscript. NI, HM, NY, MK, SK, and MM were involved provided ESCC cases. KU and NM were involved in RNA analysis. TH, TN and MN were the pathologist and evaluated the histopathology of the cases. AO was involved in the RNA analysis and additional technical assistance. MK, YK, PAG, and GJG participated in luciferase reporter assays. TN and SK coordinated the study and drafted the manuscript. SY helped in drafting the manuscript. All authors read and approved the final manuscript.
